# Supplementary figures and images for: Super-resolution imaging of RAD51 and DMC1 in DNA repair foci reveals dynamic distribution patterns in meiotic prophase
Source: PLoS Genet. 2020 Jun 5;16(6):e1008595. doi: 10.1371/journal.pgen.1008595 (PMC7310863; doi:10.1371/journal.pgen.1008595)

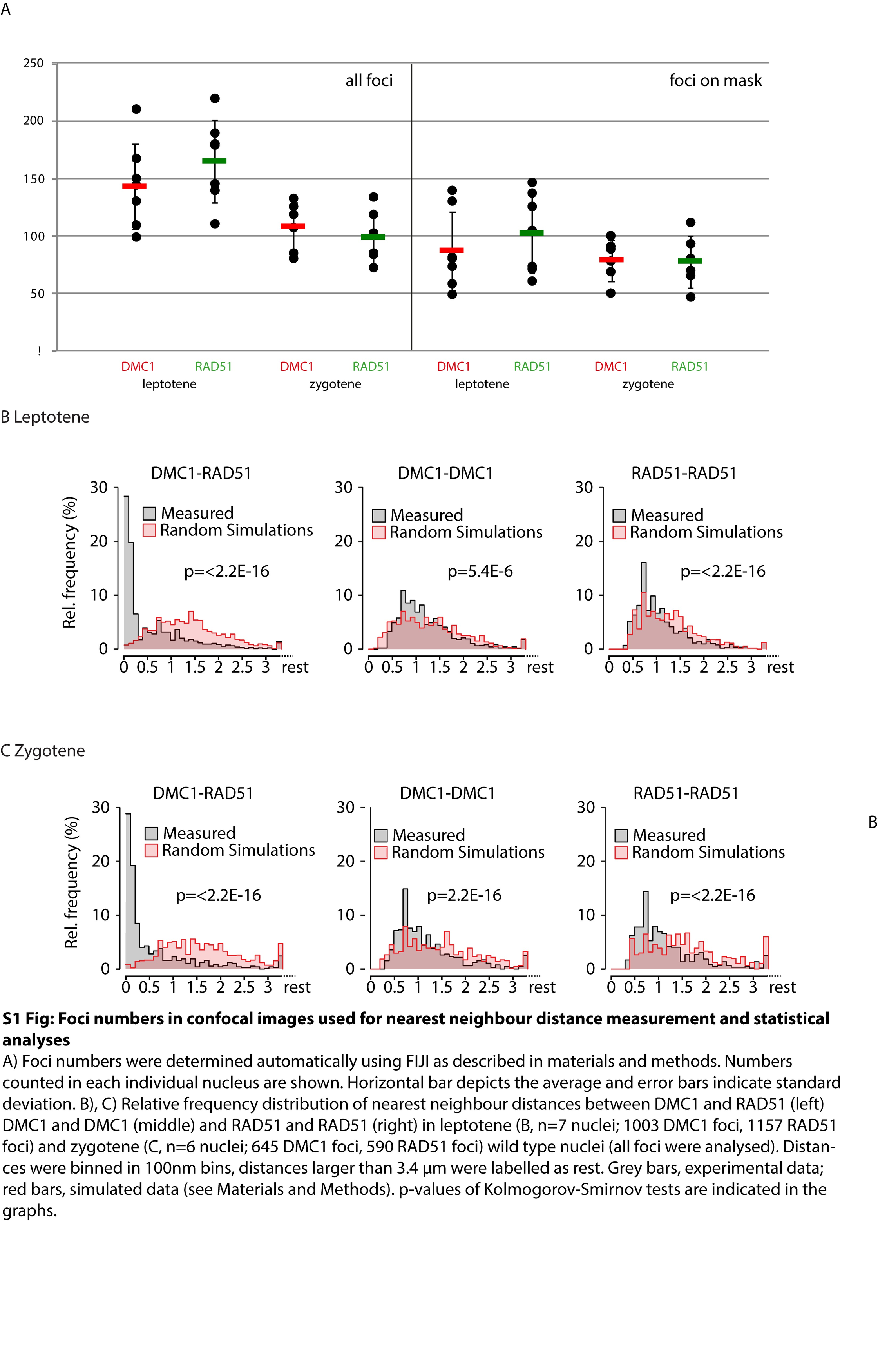

Supplement: S1 Fig — Foci numbers in confocal images used for nearest neighbour distance measurement and statistical analyses (A) Foci numbers were determined automatically using FIJI as described in materials and methods. Numbers counted in each individual nucleus are shown. Horizontal bar depicts the average and error bars indicate standard deviation. B), C) Relative frequency distribution of nearest neighbour distances between DMC1 and RAD51 (left) DMC1 and DMC1 (middle) and RAD51 and RAD51 (right) in leptotene (B, n = 7 nuclei; 1003 DMC1 foci, 1157 RAD51 foci) and zygotene (C, n = 6 nuclei; 645 DMC1 foci, 590 RAD51 foci) wild type nuclei (all foci were analysed). Distances were binned in 100 nm bins, distances larger than 3.4 μm were labelled as rest. Grey bars, experimental data; red bars, simulated data (see Materials and Methods). p-values of Kolmogorov-Smirnov tests are indicated in the graphs. (TIF) [file pgen.1008595.s001.tif]

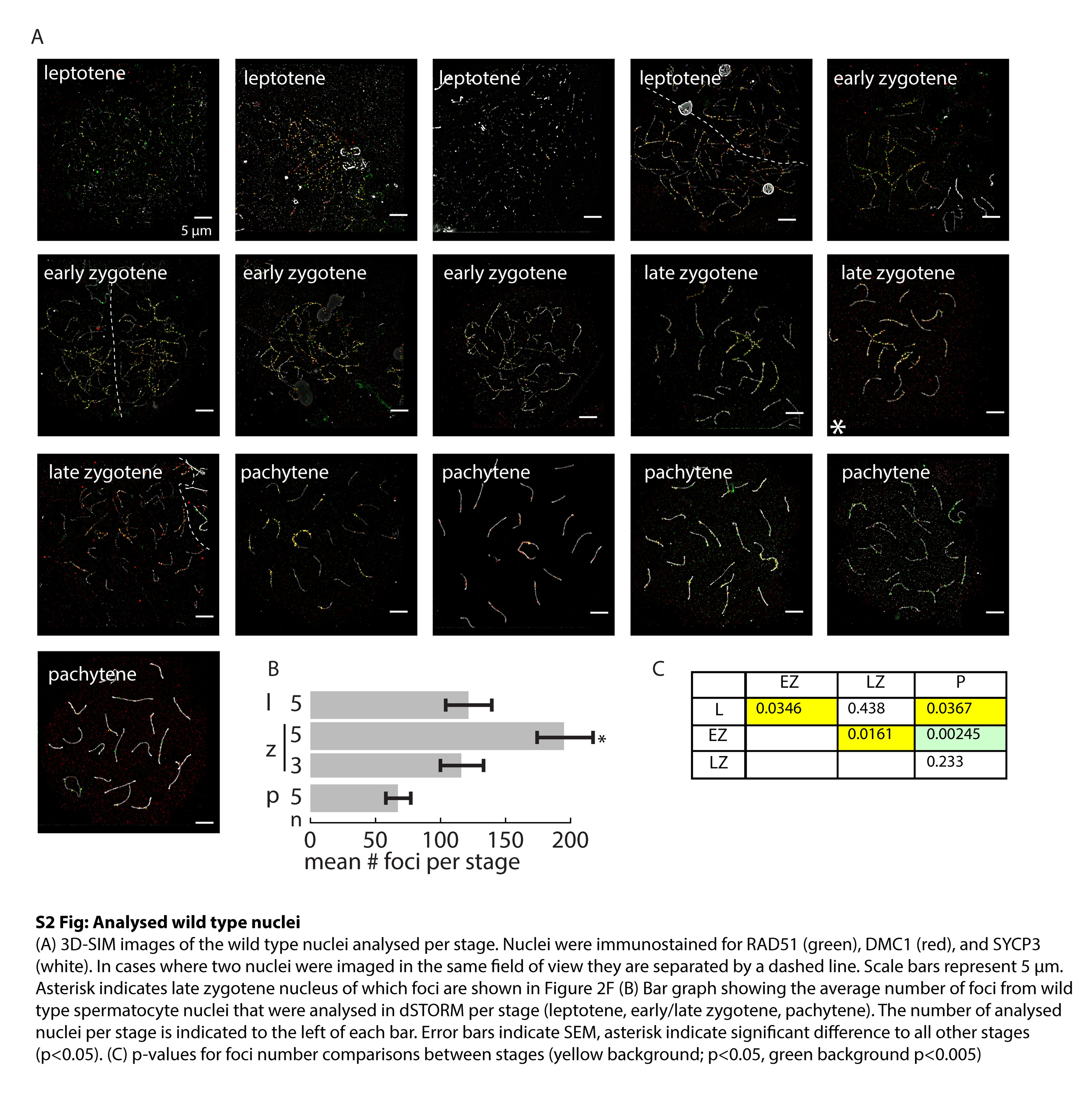

Supplement: S2 Fig — Analysed wild type nuclei (A) 3D-SIM images of the wild type nuclei analysed per stage. Nuclei were immunostained for RAD51 (green), DMC1 (red), and SYCP3 (white). In cases where two nuclei were imaged in the same field of view they are separated by a dashed line. Scale bars represent 5 μm. Asterisk indicates late zygotene nucleus of which foci are shown in Fig 2F (B) Bar graph showing the average number of foci from wild type spermatocyte nuclei that were analysed in dSTORM per stage (leptotene, early/late zygotene, pachytene). The number of analysed nuclei per stage is indicated to the left of each bar. Error bars indicate SEM, asterisk indicate significant difference to all other stages (p<0.05). (C) p-values for foci number comparisons between stages (yellow background; p<0.05, green background p<0.005). (TIF) [file pgen.1008595.s002.tif]

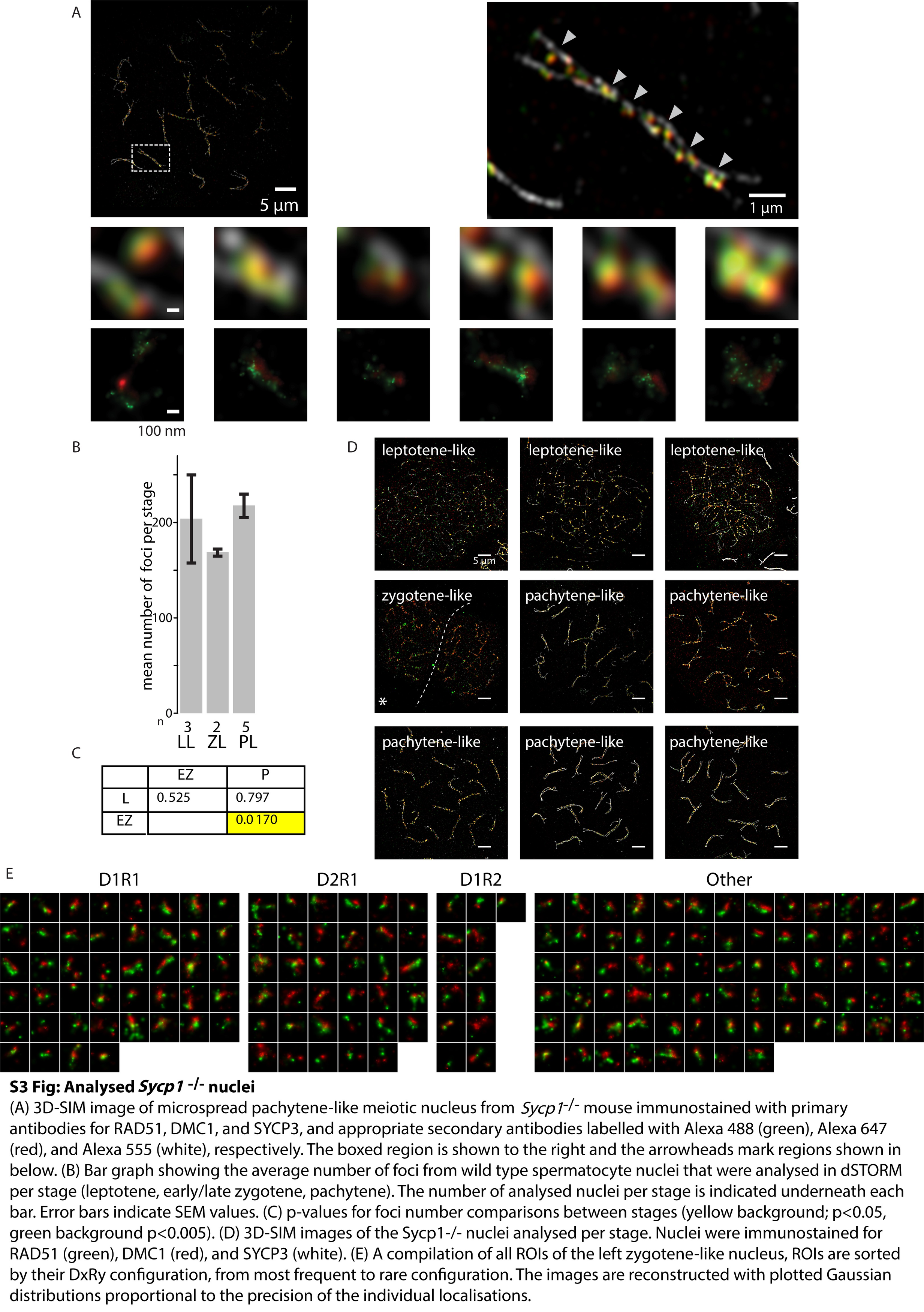

Supplement: S3 Fig — Analysed Sycp1-/- nuclei (A) 3D-SIM image of microspread pachytene-like meiotic nucleus from Sycp1-/- mouse immunostained with primary antibodies for RAD51, DMC1, and SYCP3, and appropriate secondary antibodies labelled with Alexa 488 (green), Alexa 647 (red), and Alexa 555 (white), respectively. The boxed region is shown to the right and the arrowheads mark regions shown below. (B) Bar graph showing the average number of foci from wild type spermatocyte nuclei that were analysed in dSTORM per stage (leptotene-like, zygotene-like, pachytene-like). The number of analysed nuclei per stage is indicated underneath each bar. Error bars indicate SEM values. (C) p-values for foci number comparisons between stages (yellow background; p<0.05). (D) 3D-SIM images of the Sycp1-/- nuclei analysed per stage. Nuclei were immunostained for RAD51 (green), DMC1 (red), and SYCP3 (white). (E) A compilation of all ROIs of the left zygotene-like nucleus shown in D (indicated with *), ROIs are sorted by their DxRy configuration, from most frequent to rare configuration. The images are reconstructed with plotted Gaussian distributions proportional to the precision of the individual localisations. (TIF) [file pgen.1008595.s003.tif]

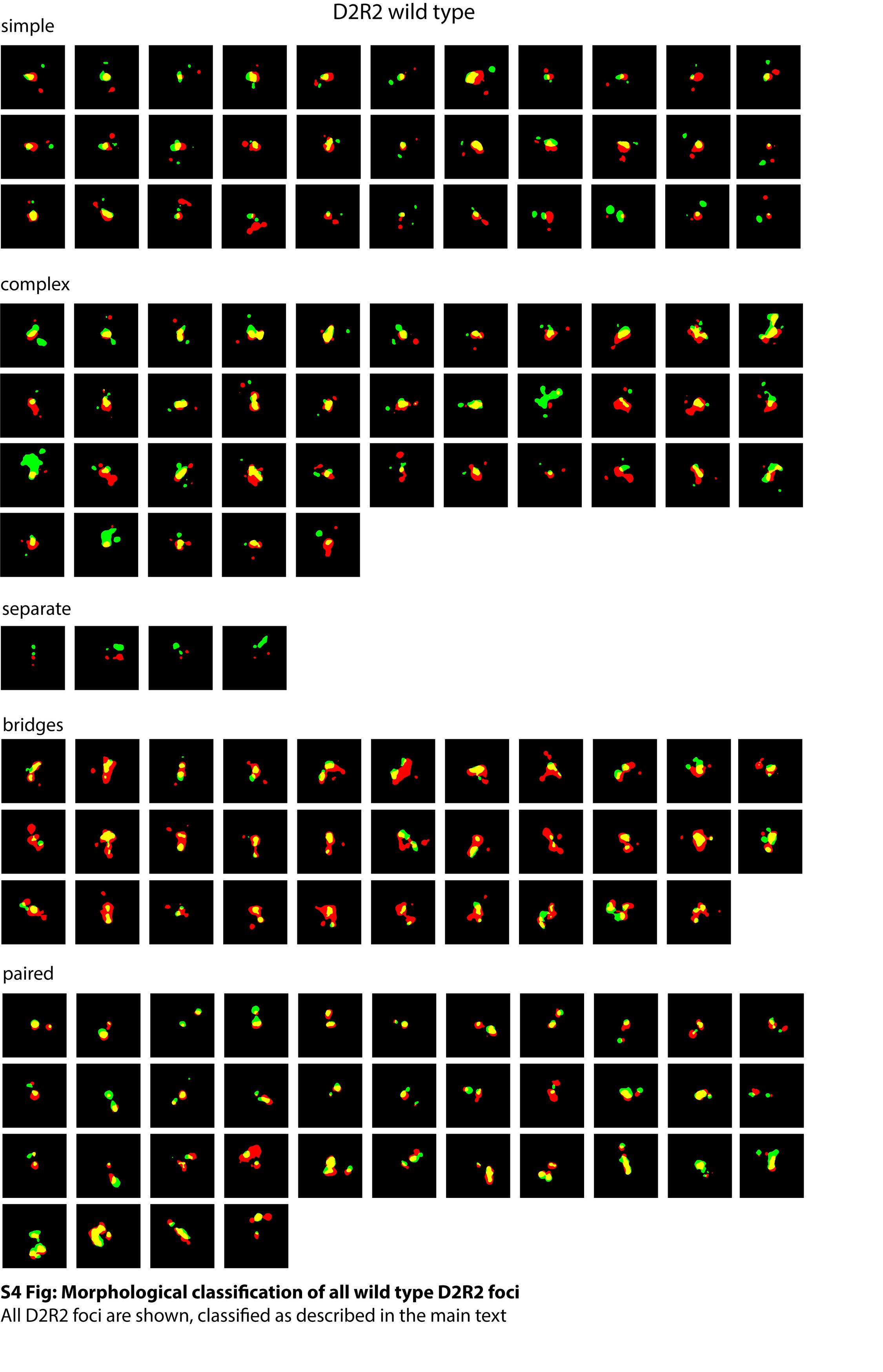

Supplement: S4 Fig — (TIF) [file pgen.1008595.s004.tif]

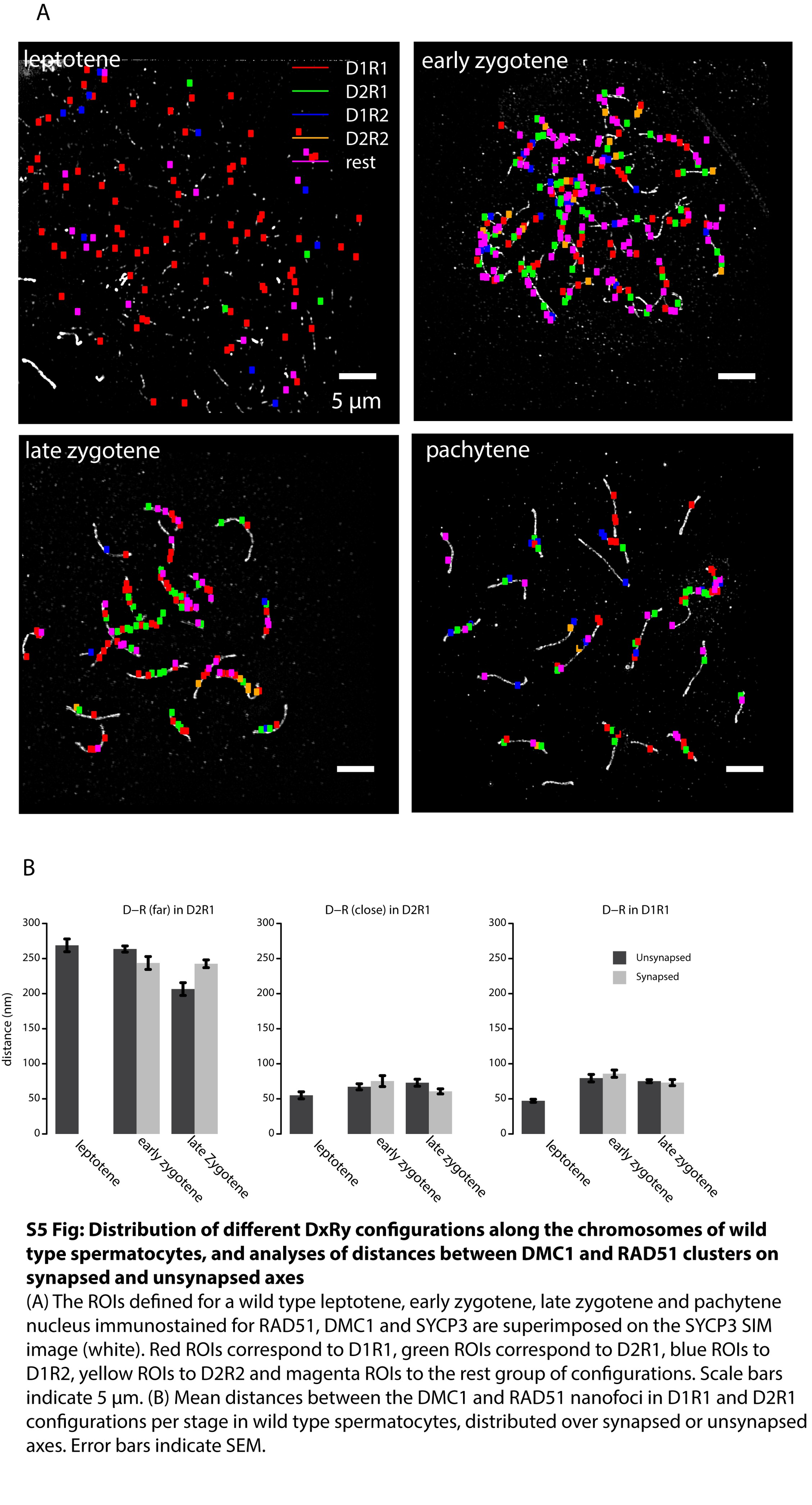

Supplement: S5 Fig — (A) The ROIs defined for a wild type leptotene, early zygotene, late zygotene and pachytene nucleus immunostained for RAD51, DMC1 and SYCP3 are superimposed on the SYCP3 SIM image (white). Red ROIs correspond to D1R1, green ROIs correspond to D2R1, blue ROIs to D1R2, yellow ROIs to D2R2 and magenta ROIs to the rest group of configurations. Scale bars indicate 5 μm. (B) Mean distances between the DMC1 and RAD51 nanofoci in D1R1 and D2R1 configurations per stage in wild type spermatocytes, distributed over synapsed or unsynapsed axes. Error bars indicate SEM. (TIF) [file pgen.1008595.s005.tif]
