# Supplementary material for: Super-resolution imaging of RAD51 and DMC1 in DNA repair foci reveals dynamic distribution patterns in meiotic prophase
Source: PLoS Genet. 2020 Jun 5;16(6):e1008595. doi: 10.1371/journal.pgen.1008595 (PMC7310863; doi:10.1371/journal.pgen.1008595)
Supplement: Attachment — Submitted filename: Response to reviewers comments.pdf [file pgen.1008595.s009.pdf]

RESPONSE TO REVIEWERS' COMMENTS

Guest Editor comments:

1) The use of the term "cluster" in place of foci in many, but not all, parts of the paper is confusing. Although I appreciate that the terms focus (singular) and foci (plural) are most frequently used to describe structures that are below the resolution limit of the microscopy method, that is not strictly the case. Given that the field is familiar with the terms 'focus" and "foci", I think it best to use those terms throughout the paper. The term "cluster" is most frequently used to describe a grouping of discrete objects and using it to describe a single contiguous region of staining, albeit composed of a cluster of epitopes, will likely be as confusing to other readers as it was to me, at first. This is particularly true because much of the paper describes clusters of foci (clusters of clusters). One can describe a given focus as being small, big, elongated etc. I would entertain a proposal to use some other term that does not have the problem that the term "cluster" has, but I think using the term "focus" is most straightforward given that it is so prominent in the literature. (Note that many papers incorrectly use the term "foci" as both singular and plural. Again, the singular is "focus.")

*We appreciate this comment, and we agree that the term cluster may be confusing since it usually refers to higher order aggregations instead of substructures. The RAD51 and DMC1 clusters we describe in our manuscript are substructures of the well-known DSB repair foci, and these binary areas do not represent a single contiguous region of staining since the density of localization events may vary within these areas. This is why we initially chose the word cluster; in referral to the clustering of localization events to a density above a set threshold within these areas. To avoid confusion because of the use of the word cluster, but also to make the distinction between the clusters and the foci that are identified at lower resolution, we have now replaced cluster with nanofocus, and explained that these represent the areas defined in the nanoscopic analysis.*

2) I was disappointed and somewhat confused by the use of a mask to eliminate foci that are not colocalized to the axial/lateral elements. This seems to me to have been a poor choice given that there are models for the homology search that invoke a searching arm that is released from the axes. One model invokes a "tentacle" long enough to search the entire volume of the nucleus and our lab's preferred model suggests a shorter tether to the axis of up to 400 nm in yeast. I imagine that examination of SIM images did not provide evidence of the focus pairs seen in other organisms with one member of each pair off the axis. If no real care was taken to discount this possibility, that is a problem. If repeating the nearest-neighbor analysis without the mask does not reveal more pairing of foci than was the case with the mask, then the mask is not a problem. HOWEVER, this analysis of the available images needs to be done and the results reported to assure the reader that important data was not lost by the method (perhaps in a supplemental figure). I also think the paper needs a better rationale as to why the mask was used, given that not all foci fall on the SC and there is no obvious reason to think that foci that do not colocalize with the SC are irrelevant to meiotic recombination.

*We agree with the editor that the use of a mask precludes the possibility of finding paired foci in cases where one focus is situated on the axes and the other focus is off the axes. However, we also felt that we needed a mask because it can be clearly seen that the foci are not randomly distributed in the nucleus, because the vast majority is clearly on the axes, and their "freedom" of localization is limited by the axial/lateral elements. So, if we would not use a mask, and would compare the distribution of foci to a random distribution, this random distribution pattern would always be very different, because these random foci could then be anywhere in the nucleus. A second reason for including only the foci on the axes is that it results in a reduction of background signal. However, we will also lose some specific signal. We tested different settings of the "noise tolerance" value for each antigen, and found that there was a rather gradual decrease in the number of foci (counting "maxima") that is detected, making the choice of the setting arbitrary in the sense that there will be always some background, and always some loss of specific signal at any setting between 60-120. We chose 90 for DMC1 and 100 for RAD51 because this seemed to be the best trade-off, and kept this constant in all subsequent analyses.*

*Still we agree that we should also perform the analyses without the mask, so we now include these analyses (revised Supplemental Figure 1). The results show that the overall colocalization between DMC1 and RAD51 (distance <300nm) is less when the mask is not used, which fits with the notion that the relative number of background foci is higher when foci in the whole nucleus are included. The DMC1-DMC1 and RAD51-RAD51 nearest neighbor distributions do not provide any indications for more close proximity pairing, the peak of distances occurring more frequently compared to the random distribution also lies between 500 and 800 nm, as we found for the foci on the mask. The results are shown in the revised Figure S1.*
*To describe the results we adapted the relevant section:*

*"To ensure nonbiased quantification of immunosignals we selected foci in leptotene and zygotene nuclei using FIJI, see Materials and Methods. We then counted the numbers of RAD51 and DMC1 foci (Supplemental Figure S1A), and used these numbers to simulate random distributions of the same number of artificially generated foci. We observed that RAD51 and DMC1 foci localisation was mostly confined to the areas in which the axial and lateral elements of the SC were forming upon visual inspection of the images. Since this feature generates a non-random organisation of the foci in the nucleus, we also used a mask to select only those foci that were located on the chromosomal axes (examples of selected foci and raw images are shown in Fig 1A, C), and used these foci numbers to also simulate random distributions along the areas covering the SYCP3 signal for each nucleus as described in Materials and Methods (see examples in Fig 1A, C). This second analyses allowed assessment of random or non-random distribution of DSB-repair foci along the axial/lateral elements. In leptotene, the use of the mask led to a reduction of 40 and 38% of the DMC1 and RAD51 foci, respectively, whereas 27% of DMC1 foci and 22% of the RAD51 foci located outside of the mask in zygotene. These "lost foci" are expected to represent background signal, as well as some sites of true RAD51 and DMC1 accumulation on DSB repair sites not localised on the axial/lateral elements. Subsequently, we determined the nearest distance between RAD51 and DMC1 foci, as well as the RAD51-RAD51 and DMC1-DMC1 distances for both types of foci selection. These analyses showed that 55 % (all foci) or 80% (on the mask), and 57% (all foci) or 67% (on the mask) of the analysed DMC1 foci had a RAD51 neighbour at a distance shorter than 300nm in leptotene and zygotene, respectively (For p-values and other statistical parameters see Supplementary Fig 1C and S2 Table), reflecting the overall colocalization. Analyses of DMC1-DMC1 and RAD51-RAD51 distances also revealed a non-random distribution (Fig 1B, Supplementary Fig 1B, C) for both the analyses performed on all foci, and the foci on the mask. Also, for both types of analyses, distances between 500 and 800 nm occurred more frequently than expected based on a random distribution. This could be explained by the fact that DSB foci are generally excluded from specific regions, such as constitutive heterochromatin and near centromeric areas, causing foci to be in closer proximity to each other than expected based on random distribution. However, the rather sharp peaks of RAD51-RAD51 and DMC1-DMC1 nearest neighbour distances around 800nm in zygotene within the DSB-foci positive SC regions, indicate additional non-random distribution."*

3) Although the scope of the paper is ok as is, the paper would be of MUCH more impact and interest if it included analysis of the average minimum distance of RAD51 and DMC1 foci to the midline of Scyp3 axes. I appreciate the near impossibility of 3-color STORM, but is it not possible to use SIM images to ask if RAD51 is closer or farther from the axis than DMC1? This question is of particular interest given the work from the Donnelly group showing that DMC1 loads 3' or RAD51 at recombination hotspots. Is DMC1, which is likely to be the active recombinase based on results from yeast and plants, held away from the axis in a manner that may facilitate pairing? It seems to me that this is an important question that the authors may have the data in hand to address.

*We thank the reviewer very much for this suggestion and we have included this analysis in the revised manuscript. For this we estimated the center of the axes by drawing a line through the SIM images of wild type zygotene and pachytene nuclei, and subsequently measured the minimal distance of the center of mass of each nanofocus to this line. We analysed the nanofoci of D1R1, D2R1 and D1R2 foci, and discriminated between the "far" and the "close" DMC1 and RAD51 foci. The results show that DMC1 is a bit further away from the axes than RAD51 when all nanofoci are taken together. The dataset is too small to see this effect for the split D1R1, D2R1, and D1R2 groups, except for the far DMC1 nanofocus in the*

*D2R1 group, which is furthest from the axes. Still it is interesting that the observations would be largely consistent with the hypothesis put forward by the guest editor. To test this hypothesis we agree that 3-color STORM and analyses of other repair components (RPA for example), and/or the DNA itself would be required, so we have only briefly discussed the possible implications.*

*New section in results:*
*"In the wild type nuclei, we next analysed the distance of the center of mass of all RAD51 and DMC1 nanofoci to a line manually drawn through the center of the SYCP3 signal in zygotene and pachytene nuclei (Fig 6A, B). When all foci were analysed together we observed that DMC1 was somewhat further away compared to RAD51 (Fig 6C, p< 0.0001, (Mann Whitney U test)). When the D1R1, D2R1 and D1R2 foci were analysed separately, a significantly larger DMC1 distance was only observed for the far DMC1 nanofocus in the D2R1 configuration (Fig 6C, p=0.0075, (Mann Whitney U test))."*

*Adaptations in discussion:*
*"Interestingly, in our analyses of the distance of RAD51 and DMC1 nanofoci to the axial/lateral elements of the SC, we observed that DMC1 nanofoci were further away from the axes than the RAD51 nanofoci. In combination with the recent CHIP-seq data, that indicate that DMC1 would be loaded more towards the 3'end of the single-stranded DNA compared to RAD51(32), and previous suggestions along these lines based on differences in biochemical properties of the two proteins (35), it might be suggested that our observations would be consistent with the hypothesis that DMC1 filaments would be held further away from the axes because of their presence at the ends of the filaments and their homology probing function. However, this hypothesis needs to be tested in more detail in future experiments that involve three-colour STORM analyses and include other components such as DNA, and/or functional tests.*
*"*

*New section in material and methods:*
*"The minimal distance from the axial/lateral elements was determined for each nanofocus in wild type zygotene and pachytene nuclei. A line was manually drawn through the middle of the SYCP3 signal in the SIM images and the shortest distance between the axis and the nanofocus was determined. Nanofoci with a distance >1µm were considered not near an axis. Pairwise comparison between the mean values of DMC1, RAD51 and the specific far nanofoci was performed using a Mann Withney U test. A p-value below 0.05 was considered a significant difference."*

4) Line 112. Capitalize the abbreviation for Saccharomyces.
*This has been corrected*

5) Line 163. Why was a diameter of 600 nm chosen to define a ROI?
*The resolution limit of standard (confocal) microscopy images is around 250nm. So, if we draw a ROI of 600nm diameter around the center of the observed focus, this would still allow most of the foci that were visibly separable to end up in two separate ROIs. At the same time, it would allow detection of the presence of paired nanofoci within a ROI at distances below the resolution limit of the confocal microscope.*

*This is now described in the main text as follows:*
*"A total dataset of 2315 manually selected foci (ROIs of 600 nm diameter circles drawn around the centre of each focus, to allow separate selection of foci at distances of >300 nm) was generated by analysis of 18 nuclei in different meiotic substages, imaged in four independent experiments (Supplemental Fig S2A-C, S1Table)."*

6) Line 166 and in many other places in the manuscript. Claim is made that: "Foci with multiple RAD51 or DMC1 clusters were also present, and were somewhat more frequent for DMC1 compared to RAD51." In such instances, if would be helpful if the authors indicated the fold difference between the two frequencies and give the P value for significance of the difference.

*We have now included all appropriate statistical analyses, also at all other locations indicated by the guest editor. This has led to some minor adaptations in Figures 3-7, and we added a supplementary Excel file with results of statistic analyses (S2 Table). We have also included p-values and information about the statistical tests used wherever appropriate in the main text.*

7) I also ask "was a similar difference seen in a least two independent repeats… in two independent experiments? One has to be particularly concerned about this given that some of the "satellite" foci are likely to be near the detection limit, such that staining efficiency could impact the results. There are a number of similar instance of claims of small differences in the paper, as mentioned below. Are have all of these observations been repeated?

*The frequency of detection of a second smaller DMC1 or RAD51 focus is reproducible between experiments: when we consider the relative DxRy frequency distribution of all foci collected in each of the four independent wild type experiments, and the two independent Sycp1 knockout experiments, we observed similar frequencies of the three main configurations for each experiment. In order to determine the variation of the observed frequencies we have taken every cell independently and calculated the average and variation (sem values are shown in the bar graph below) between the different cells that were recorded at multiple days. Between the independent experiments at different days we observe a similar distribution of nanfoci composition. This figure is not included in the manuscript, but the source data are in Table S1.*

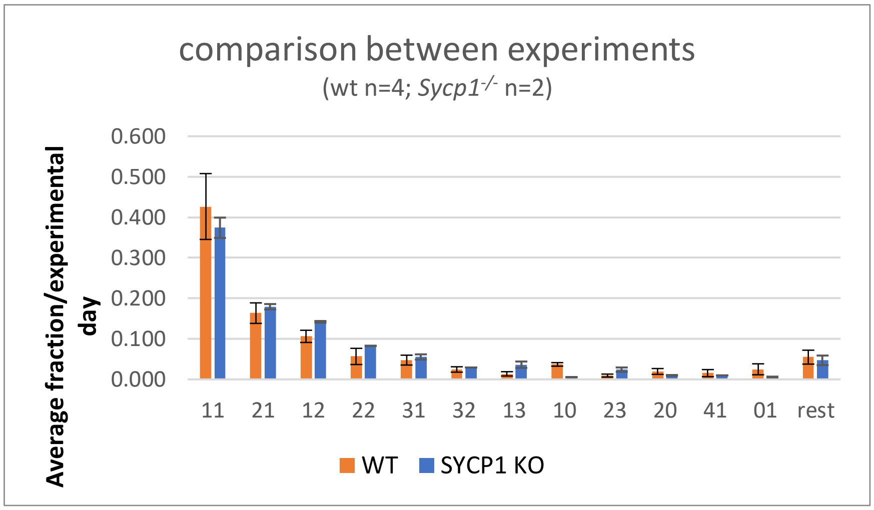


8) Line 193. "Domain" is a potentially confusing synonym. "Focus" would be a more clear.

*We have adapted this according to the terminology of foci and nanofoci that we now defined as explained in our reply to comment 1*

9) Line 205. Are the structures referred to as bridges always elongated or is the definition simply based on the relative position of the RAD51 focus in between two DMC1 foci (or vice versa)?

*The definition of a bridged structure is indeed based on the latter: for D2R1: the RAD51 nanofocus overlaps partially with each of the two DMC1 nanofoci (and vice versa for the D1R2)*

10) Line 211. It is not clear to me what distinction is being made from paired verses unpaired D2R2 foci. Aren't D2R2 foci paired by definition?

*We considered the following as paired: a RAD51 and DMC1 nanofocus that partially overlap with each other, and a second similar partially overlapping RAD51-DMC1 structure, but separate from the other. In nonpaired D2R2 foci, there can for example be a D2R1 bridged structure with an R satellite, or D1R2 bridge with a D satellite, or a very complex structure whereby all nanofoci would be connected in one structure. We have tried to more clearly describe our definition of paired D2R2 foci in the revised text:*

*"Special attention was given to the occurrence of what could be considered as paired configurations, defined as a separated twin set of partially overlapping RAD51 and DMC1 nanofoci (Fig 3A: "paired" and Fig S4)."*

11) Line 246. It is confusing that this paragraph switches from description of "minimum distance" to average distance and also that 70 nm is the typical distance of each focus center to the center of mass AND the typical distance between centers of RAD51 and DMC1 foci. This could be more clearly explained.

*We apologize for the confusion, we adapted the text as follows:*

*"Interestingly, minimum distances coherently clustered at approximately 70 nm (wild type/Sycp1$^{-/-}$;68.4±1.2sem/75.8±1.1sem) for all analysed foci configurations in wild type and Sycp1 knockout nuclei. Thus, almost all foci that contain more than one RAD51 and/or DMC1 nanofocus, contain at least one RAD51 and one DMC1 nanofocus in close proximity to each other, the minimal distance averaging at ~70nm, (Figure 5A)."*

12) Line 262 It is stated that "the measured larger distance between the two DMC1 or RAD51 clusters in the D2R1 and D1R2 configurations can be interpreted as more spatial separation." This seems like a ridiculous statement. Is there any way two things that are farther apart would <u>not</u> be interpreted to have more special separation? Am I missing something?

*The distance is measured between the center of mass of the nanofoci, thus, if one of these becomes more elongated, the distance may increase, but the size of the area of the nanofoci that overlaps between RAD51 and DMC1 may still be the same, so they need not be spatially separated in that sense. Since we observed that the far nanofocus was small, we could distinguish between these two possibilities and conclude that the larger distance indeed results from more spatial separation. We have adapted the text to make this more clear as follows:*

*"This observation of asymmetry allowed us to define close and far nanofoci in both the D2R1 and the D1R2 configurations. Interestingly, we observed a large close nanofocus and a small far nanofocus irrespective of whether two RAD51 or two DMC1 nanofoci were present (Figure 5C-E). Thus, the measured larger distance between the two DMC1 or RAD51 nanofoci in the D2R1 and D1R2 configurations can be interpreted as more spatial separation, and is not caused by a second structure that is very spread out."*

13) Line 340 How can a simulated structure be spurious? Does this mean that the R focus is arranged in such a way that it divides a single D focus in two? -ie not "detecting an additional cluster" but rather the overlap of a single focus by a narrower 51 focus makes it look like 51 is separating two distinct DMC1 foci?
*Indeed it seems odd that a simulated structure can be spurious however this is explainable by the used model. The model is generated by different random distributions of points in the 3D volume. One of these distributions represents noise and is present throughout the 3D volume. When viewed in 2D, these noise distributions can form a structure just above background by chance. In other words, sometimes the simulated noise does cluster which is a known feature of random distributions, this effect is enhanced by the flattening of 3D points into a 2D projection thus increasing the chance of spurious structures. We have explained this more clearly as follows:*

*"Interestingly, around 15% of the simulated D2R1 configurations in a three-dimensional space are represented as D1R1 in the two-dimensional representations, and also a small fraction of D3R1 and D2R2 configurations were observed for simulated D2R1s. This is most likely caused by situations whereby noise detections cluster and rise just above the background, resulting in detection of an additional nanofocus."*

14) Line 343. I have not seen the phrase "degree of freedom for the angle" used before, which could reflect my ignorance! Is this alternative statement accurate? "the angle of rotation that provided the best fit to the experimental data set was…" If not, a better explanation of the manipulation used to give the best fit is needed for those, like me, with a limited math background.

*The model used does not specify a fixed angle between the RAD51 axis and the line between the two DMC1 nanofoci, instead a maximum angle is specified. Generated models have a random angle assigned between 0° and the value specified by the parameter α. We don't expect the D2R1 structure to be completely rigid, since the filaments they most likely represent are expected to be dynamic and may also change their dynamic behavior as prophase progresses. So we anticipate to fix the structures in different possible configurations. Our model reflects this by not generating the same structure but simulating a probing structure. Our simulated data and generated consensus patterns indicate this model approaches the data best. We adapted the text to make this more clear:*

*"Next, we simulated a 3D model of D2R1 configurations. In short, the model consists of three randomly generated structures reflecting the two DMC1 nanofoci and a single RAD51 nanofocus, and a random noise component. To find the best fitting model we varied the length (σ) of the RAD51 nanofocus, and the maximum angle (α) between the line connecting the two DMC1 nanofoci and the line connecting the RAD51 nanofocus to the close DMC1 nanofocus. The fixed length and variable angle reflect a possible homology searching structure as described in more detail in Materials and Methods (Fig 9A)."*

15) Line 374. Here I think it is worth comparing the results described here to those we described for the equivalent experiment in budding yeast, where foci in yeast are a bit shorter. It is also worth mentioning that we too concluded that recombination filaments are likely shorter than ssDNA tracts.

*We have included this comparison in the revised version of the Discussion section in the manuscript*

16) Line 534. The term maxima may confuse people not familiar with quantitative microscopy. If there is always one maxima per focus you could just say something like "the number of foci detected in a given image determined the number of simulated foci randomly projected in 3 dimensions to create a simulated image." I also note that this section has a lot of technical language, but lacks specific explanation for why certain procedures were necessary—i.e. what they contributed to the validity/utility of the simulated images. I take it that the manipulations were intended to mimic characteristics of the imaging system, but I think it would be helpful to state that directly.

*We have first explained better what the result is of employing the "find maxima" function in FIJI" as follows:*

*"The confocal images were analysed to determine the distribution of RAD51 and DMC1 along the synaptonemal complexes by measuring the nearest neighbour distances. Single nuclei were manually segmented, next DMC1 and RAD51 foci were detected with the ImageJ function "Find Maxima", and a noise tolerance value of 90 (DMC1) and 100 (RAD51). Each detected "maximum" represents a single pixel with associated (x,y) coordinates, and thus each focus is thereby represented by a single x and y value in the image."*

*In addition, we have tried to provide a more comprehensive description of the random simulations:*

*"Simulated images were created by generating random single pixel foci (maxima) until the same number of foci were generated within the nuclear area or within the boundaries of the SYCP3 signal as the number of foci that had been counted in the nucleus, or within the mask, respectively. To achieve this, foci were generated randomly in the image, and those localising outside the nuclear or masked boundary were discarded, and remaining positions were marked. This was continued until the number of marked positions corresponded to the required number of foci. To correct for the diffraction limited signal of a confocal microscope, the random image was blurred with a Gaussian filter with a sigma value of 0.11 µm. This sigma value is approximately the standard deviation of the confocal microscope (FWHM = $2\sqrt{2\,ln\,2}\,\sigma \approx 2.355\,\sigma$ (Weisstein, 2002)). This results in all foci having precisely the same degree of blurring. To add simulated variation between foci, simulated shot noise was added by adding a value of 5 to the entire image, and subsequently adding a random value between +/- the square root of the intensity of each individual pixel. This image was then processed in the same way as the confocal images. 50 random simulations were performed for each nucleus."*

*For the reviewers we add here a simplified display of the creation of random spots in the simulation:*

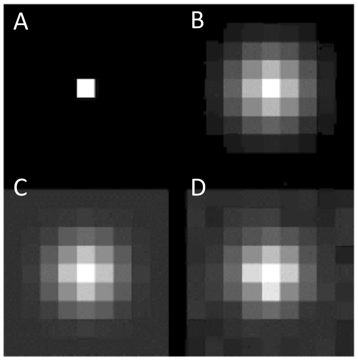


***(A)*** *A single pixel is projected onto the image.* ***(B)*** *A Gaussian filter with a standard deviation of 0.11 µm is applied over the image.* ***(C)*** *To the image an intensity of 5 is added.* ***(D)*** *Every individual pixel has a value between -1 and 1 times the square root of its intensity added to simulate random noise.*

17)Line 566. It is a relatively straightforward experiment to distinguish between the two possibilities listed, i.e a quantitative western blot of titrated amount of protein. One simply needs a little pure RAD51 and pure DMC1. Many labs share these reagents, including ours. This would allow the authors to estimate the relative amounts of each protein in the structures they detect. I will not require that this be done for publication, but it easily could be done and such information would add substantially to the paper.

*We thank the reviewer for this suggestion, and we think it could be a very useful experiment to assess the relative affinities of the two antibodies in detecting the purified protein on a western blot. However, we feel that this will not provide a definite answer to discriminate between the relative affinities of the RAD51 and DMC1 antibodies to their target proteins when these are in a chromatin environment and have been fixed with paraformaldehyde, and antigen accessibility also becomes an important factor that may influence detection efficiency. Still, we agree with the importance of this issue. One future approach could be to immobilize a known amount of the proteins on single stranded DNA on a coverslip used for dSTORM analyses, followed by similar processing steps as those that accompany the generation of spread nuclei, but this would require substantial optimization experiments and is outside the scope of the current manuscript.*

Reviewer comments:
1) Improved resolution of figure panels:
The resolution of many figure panels in the pdf provided was of poor quality. This was true both for images and for graphs.

*We have now included the high resolution images and improved the quality of the figures.*

2) Frequency vs number: The term “frequency” appears to be used in multiple different ways in different parts of the manuscript, leading to some confusion, especially in cases where the numbers of foci present change between stages. Examples where this is a problem will be discussed below, but care should be taken throughout the manuscript to be precise in use of language to avoid such confusion.

*We apologize for this confusion and have edited all the relevant text, also in accordance with the suggestions related to this issue below. For many figures we now include graphs displaying numbers of foci in addition to the graphs representing the fraction of foci, and replaced relative frequency with fraction, and frequency with number wherever appropriate.*

3) Basis for staging of nuclei for Sycp1 mutant: Whereas staging of nuclei from the WT is straightforward based on the state of synapsis, this becomes challenging in a mutant in which synapsis does not occur. In order to make strong statements regarding progression and timing in the context of the Sycp1 mutant, confidence in staging is crucial. Please indicate how stages were assigned for the mutant, and the degree of confidence in these assignments.

*All the nuclei that were analysed are in Supplemental Figure 3. Leptotenes were defined as nuclei whereby axial element formation was incomplete. In zygotene-like nuclei, axial elements were complete and pairing was initiated. In pachytene-like nuclei the majority of pairs were aligned or in close proximity to each other.*

4) L 51 clarify wording: repair sites often contain adjacent or overlapping single large clusters of the two proteins…
L54 synapsis (instead of pairing)
L79 suggesting (instead of indicating)
L101 use of the D1R1 terminology that is not yet introduced

*These points have all been addressed in the revised manuscript*

5) Figure 1 and related text:
Please clarify how the random distributions were simulated. From the Methods, it is clear how the simulated images were treated, processed and analyzed, but it was not clear how the initial random distributions were actually generated in the first place. E.g. was an existing ImageJ plug-in used, or was there a custom script used to do this? This information will be helpful for other researchers in the field that want to do similar analyses.

*We have adapted the relevant materials and methods section to make it more clear, and easily reproducible by researchers familiar with FIJI. See also our response to comment 16 of the guest editor. In general, random images were generated by the following pseudocode:*

```
for(numberOfMaxima){
do{
    x = random()*widthOfImage;
    y = random()*heightOfImage;
  }while( !inMask(x,y)  );
}
```

For the Figure, please increase font sizes wherever possible, including making it easier to see “leptotene” and zygotene”. Move P values from the supplement to the main figure, putting them under each relevant graph.

*We have adapted this in accordance with the reviewers suggestion.*

6) FigS1: not clear why the D values as defined were chosen to be provided- this parameter seems rather uninformative for capturing important features of the data distribution.

*The D-value is a general parameter that is used in Kolmogorov-Smirnov comparisons of data distributions, and is informative since it provides some information regarding the degree of difference in the data distribution, where the p value indicates the statistical significance of the difference, independent of whether the difference is large or small.*

7) Figure 2 and related text:
How were foci manually selected (what criteria)? How were the ROIs selected (centered on what)? How was the size of the ROIs chosen?

*Foci were selected if a RAD51 or DMC1 focus presence was visibly recognized as being above background in the SIM images. Then the ROI was generated around the center estimated on a composite image showing both RAD51 and DMC1 signals.*

8) Fig 2G x-axis label "Number of clusters in ROI"

*This has now been adapted and reads "number of nanofoci in ROI"*

9) P9: Any claims about differences in the frequencies of different focus categories should be backed up statistically.

*We have added results of statistic analyses in all relevant instances where we describe observed differences. Details of statistics methods are described in the Materials and Methods section and detailed results of different comparisons for data in the different figures are now in Supplementary Table S2. See also our response to the Guest Editors' comment 6.*

10) Figure 3 and related text:
P9-10: Again, claims are made regarding classes that increase during meiotic progression in WT or in the Sycp1 mutant; need to show statistical support to make these claims.
Also, this is where it begins to be relevant and important to display the information in two different ways, so that BOTH the FRACTION of foci that are of each given class and the (per nucleus average) NUMBERS of foci of each class at a given stage are represented. This is necessary for thinking reasonably about whether it is appropriate to consider one class transitioning to another as prophase progresses. It may be worth considering stacked bar graphs as a useful way for representing both of these points, representing each category with a different color to make it easier to compare the distributions for each stage side by side. Here, the FRACTION graph uses the "parts of a whole" approach, totaling to 100%, while in the average NUMBERS graph the totals would be different for each stage. This would be especially helpful in thinking about comparisons between WT and Sycp1 mutant, where the numbers of foci present at the different stages are very different.

*We would like to point out that Figure 3 and 4 represent different results. In figure 3 we have manually classified foci based on shapes, not on the number of RAD51 and DMC1 nanofoci. We have adapted the main text to make the distinction between this analysis and the subsequent computational analyses on*

*the D1R1, D2R1 and D1R2 foci clearer. We have now included statistical analyses of these data and report average values per nucleus and sem values and these are not compatible with generating stacked bars. We have added the graphs with absolute average numbers of the different classes of the different meiotic stages, and taken care to clearly refer to numbers and fraction wherever this is appropriate.*

11) Figure 4 and related text:
As presented, I found this figure very confusing, in part because initially I wasn't quite sure what you meant by "absolute frequency", "average frequency" and "relative frequency". I ended up concluding that this was trying to represent the same things I discussed above for Figure 3 (i.e. fraction in each class, vs average number [per nucleus] in each class). This was confusing in part because of the axis labels and in part because only the foci in these three classes were being considered, which may be problematic for thinking about progression, as discussed above.
If the data in Fig 3 are presented in stacked bar graphs as discussed above, it is likely that this figure will be unnecessary, as the information will already be represented in a way that makes stage to stage comparisons easier and more intuitive.
The only piece of information that would not be represented already is 4C.

*As described above, this analysis is essentially different from the analyses performed for Figure 3. We have labeled the Y-axes more clearly (numbers of foci versus fraction of foci),and show the results now as average values per nucleus and include the appropriate error bars, which makes the stacked representation less suitable.*

12) L 235-6: reword (and provide p-value), as the current sentence construction is inaccurate, since all classes of foci occur preferentially on unsynapsed axes (as opposed to synapsed axes).

*This analysis is actually more complicated than it seems, and we have repeated the analyses after rethinking the whole rationale. In this analysis we determine the fraction of all foci that is on unsynapsed versus synapsed axis in that nucleus, and compare this to the fraction of the D1R1, D2R1 and D1R1 foci on the synapsed versus unsynapsed axes in that same nucleus, and performed this for all zygotene nuclei. If more than 50% of all foci localize on unsynapsed axes this does not necessarily mean that all foci preferentially localize on unsynapsed axes since it also depends on the degree of synapsis in that nucleus, and this parameter varies considerable in each cell. Therefore we have performed this paired analyses for each nucleus, and performed the appropriated statistical analyses (two-sided paired t-test). The figure has been adapted and the results are described more comprehensively as follows:*

*"Next, we determined the fraction of foci that was on synapsed versus unsynapsed axes for each wild type zygotene nucleus and compared this to the distribution of D1R1, D2R1, and D1R2 foci in the same nuclei. Interestingly, compared to the distribution of all foci, the D1R1 configuration was slightly enriched on synapsed axes while the D1R2 configuration localized preferentially on unsynapsed axes in wild type zygotene nuclei, (p = 0.017, p= 0.037 respectively, two-sided paired t-test Fig 4E, S2 Table)."*

*The Materials and Methods section has also been adapted:*

*"To test differences in the prevalence of all, versus D1R1, D2R1 and D1R2 configurations on synapsed versus unsynapsed axis (Fig4E) a paired t-test was used. A paired test was used since the overall fraction of foci on synapsed axis will depend on the meiotic progression of the individual cells (S2 Table). "*

13)Figs 6 and 7:
Include legend to indicate the use of color in the quadrant maps.

*The legend for the use of colour is now included in the figures.*

14) Fig 8:
The graphs in C are so tiny and so blurry that I didn't even notice the blue and yellow dots until I read the figure legend. Increase size of graphs, increase relative size of the data dots, and indicate what they represent on the figure itself.

*This has been adapted in accordance with the suggestions.*

15) Discussion:
L385: Here should include reference to the C. elegans data on paired RAD51 foci.

*The observation on C. elegans RAD51 foci pairing has now been included in this part of the discussion.*

L426: "… increase in both the number and fraction of foci in the D1R1 configuration…" . Also in this section, consider how you are using the term frequency, as discussed above.

*We have adapted the wording to make it clearer*

Methods:
Nearest neighbor: what fraction of foci were outside the mask? What was the width of the mask?

Random simulation: see above regarding Fig 1

*We have now performed the nearest neighbor analyses with and without the mask. In leptotene the use of the mask led to a reduction of 40 and 38% of the DMC1 and RAD51 foci, respectively, whereas 27% of DMC1 foci and 22% of the RAD51 foci located outside of the mask in zygotene. See also our response to comment 2 of the guest editor and changes listed there. Regarding the random simulation, we have adapted the relevant materials and methods section to make it clearer, and easily reproducible by researchers familiar with FIJI. See also our response to comment 16 of the guest editor.*
