# Supplementary material for: Super-resolution imaging of RAD51 and DMC1 in DNA repair foci reveals dynamic distribution patterns in meiotic prophase
Source: PLoS Genet. 2020 Jun 5;16(6):e1008595. doi: 10.1371/journal.pgen.1008595 (PMC7310863; doi:10.1371/journal.pgen.1008595)
Supplement: Attachment — Submitted filename: Willy Baarends rev..docx [file pgen.1008595.s008.docx]

This is an interesting paper describing the structures formed by two recA family members, RAD51 and DMC1, during meiotic prophase in mouse spermatocytes. Unike observations in yeast and worms, the results seem to indicate that only a single major staining focus forms at each site of recombination rather than a pair of RAD51-DMC1 co-foci. This suggests that rather than one complex forming on each end of a DSB, there is either asymmetric loading or some other process that accounts for the observed difference. In addition to a single large staining focus for RAD51 and for DMC1, there are sometimes additional small foci of either DMC1 or RAD51 associated with the large foci. The paper also examined a mutant defective in forming the central region of the SC and identifies some minor effects of this mutant on the classes of DMC1-RAD51 structures observed. The work is meticulously done and the data is thoughtfully interpreted. Although the new insight from the work is somewhat limited, the observations will no doubt be of interest to readers of *PLoS Genetics* who study meiosis and recombination. I am therefore in favor of accepting the manuscript for publication provided a number of concerns about the manuscript are addressed. These are listed below.

The use of the term “cluster” in place of foci in many, but not all, parts of the paper is confusing. Although I appreciate that the terms focus (singular) and foci (plural) are most frequently used to describe structures that are below the resolution limit of the microscopy method, that is not strictly the case. Given that the field is familiar with the terms ‘focus” and ”foci”, I think it best to use those terms throughout the paper. The term “cluster” is most frequently used to describe a grouping of discrete objects and using it to describe a single contiguous region of staining, albeit composed of a cluster of epitopes, will likely be as confusing to other readers as it was to me, at first. This is particularly true because much of the paper describes clusters of foci (clusters of clusters). One can describe a given focus as being small, big, elongated etc. I would entertain a proposal to use some other term that does not have the problem that the term “cluster” has, but I think using the term “focus” is most straightforward given that it is so prominent in the literature. (Note that many papers incorrectly use the term “foci” as both singular and plural. Again, the singular is “focus.”)

I was disappointed and somewhat confused by the use of a mask to eliminate foci that are not colocalized to the axial/lateral elements. This seems to me to have been a poor choice given that there are models for the homology search that invoke a searching arm that is released from the axes. One model invokes a “tentacle” long enough to search the entire volume of the nucleus and our lab’s preferred model suggests a shorter tether to the axis of up to 400 nm in yeast. I imagine that examination of SIM images did not provide evidence of the focus pairs seen in other organisms with one member of each pair off the axis. If no real care was taken to discount this possibility, that is a problem. If repeating the nearest-neighbor analysis without the mask does not reveal more pairing of foci than was the case with the mask, then the mask is not a problem. HOWEVER, this analysis of the available images needs to be done and the results reported to assure the reader that important data was not lost by the method (perhaps in a supplemental figure). I also think the paper needs a better rationale as to why the mask was used, given that not all foci fall on the SC and there is no obvious reason to think that foci that do not colocalize with the SC are irrelevant to meiotic recombination.

Although the scope of the paper is ok as is, the paper would be of MUCH more impact and interest if it included analysis of the average minimum distance of RAD51 and DMC1 foci to the midline of Scyp3 axes. I appreciate the near impossibility of 3-color STORM, but is it not possible to use SIM images to ask if RAD51 is closer or farther from the axis than DMC1? This question is of particular interest given the work from the Donnelly group showing that DMC1 loads 3’ or RAD51 at recombination hotspots. Is DMC1, which is likely to be the active recombinase based on results from yeast and plants, held away from the axis in a manner that may facilitate pairing? It seems to me that this is an important question that the authors may have the data in hand to address.

Line 112. Capitalize the abbreviation for Saccharomyces.

Line 163. Why was a diameter of 600 nm chosen to define a ROI?

Line 166 and in many other places in the manuscript. Claim is made that: “Foci with multiple RAD51 or DMC1 clusters were also present, and were somewhat more frequent for DMC1 compared to RAD51.” In such instances, if would be helpful if the authors indicated the fold difference between the two frequencies and give the P value for significance of the difference. I also ask “was a similar difference seen in a least two independent repeats… in two independent experiments? One has to be particularly concerned about this given that some of the “satellite” foci are likely to be near the detection limit, such that staining efficiency could impact the results. There are a number of similar instance of claims of small differences in the paper, as mentioned below. Are have all of these observations been repeated?

Line 187 see comment re 166.

Line 193 see comment re 166.

Line 193. “Domain” is a potentially confusing synonym. “Focus” would be a more clear.

Line 205. Are the structures referred to as bridges always elongated or is the definition simply based on the relative position of the RAD51 focus in between two DMC1 foci (or vice versa)?

Line 211. It is not clear to me what distinction is being made from paired verses unpaired D2R2 foci. Aren’t D2R2 foci paired by definition?

Line 246. It is confusing that this paragraph switches from description of “minimum distance” to average distance and also that 70 nm is the typical distance of each focus center to the center of mass AND the typical distance between centers of RAD51 and DMC1 foci. This could be more clearly explained.

Line 262 It is stated that “the measured larger distance between the two DMC1 or RAD51 clusters in the D2R1 and D1R2 configurations can be interpreted as more spatial separation.” This seems like a ridiculous statement. Is there any way two things that are farther apart would not be interpreted to have more special separation? Am I missing something?

Line 265. See comment re 166.

Line 284, 288. See comment re line 166.

Line 340 How can a simulated structure be spurious? Does this mean that the R focus is arranged in such a way that it divides a single D focus in two? -ie not “detecting an additional cluster” but rather the overlap of a single focus by a narrower 51 focus makes it look like 51 is separating two distinct DMC1 foci?

Line 343. I have not seen the phrase “degree of freedom for the angle” used before, which could reflect my ignorance! Is this alternative statement accurate? “the angle of rotation that provided the best fit to the experimental data set was…” If not, a better explanation of the manipulation used to give the best fit is needed for those, like me, with a limited math background.

Line 374. Here I think it is worth comparing the results described here to those we described for the equivalent experiment in budding yeast, where foci in yeast are a bit shorter. It is also worth mentioning that we too concluded that recombination filaments are likely shorter than ssDNA tracts.

Line 534. The term maxima may confuse people not familiar with quantitative microscopy. If there is always one maxima per focus you could just say something like “the number of foci detected in a given image determined the number of simulated foci randomly projected in 3 dimensions to create a simulated image.” I also note that this section has a lot of technical language, but lacks specific explanation for why certain procedures were necessary—i.e. what they contributed to the validity/utility of the simulated images. I take it that the manipulations were intended to mimic characteristics of the imaging system, but I think it would be helpful to state that directly.

Line 566. It is a relatively straightforward experiment to distinguish between the two possibilities listed, i.e a quantitative western blot of titrated amount of protein. One simply needs a little pure RAD51 and pure DMC1. Many labs share these reagents, including ours. This would allow the authors to estimate the relative amounts of each protein in the structures they detect. I will not require that this be done for publication, but it easily could be done and such information would add substantially to the paper.

I agree with the comments of the other reviewer regarding data presentation and will not repeat those comments here.
