# Supplementary material for: Super-resolution imaging of RAD51 and DMC1 in DNA repair foci reveals dynamic distribution patterns in meiotic prophase
Source: PLoS Genet. 2020 Jun 5;16(6):e1008595. doi: 10.1371/journal.pgen.1008595 (PMC7310863; doi:10.1371/journal.pgen.1008595)
Supplement: Attachment — Submitted filename: Slotman et al_re-revised_Response to reviewer comments.pdf [file pgen.1008595.s010.pdf]

We thank the reviewers for their comments and suggestions to our resubmitted manuscript. Below we respons in detail.

Response to associate editor's comments

Line126 "selected foci in leptotene and zygotene" do you mean "selected nuclei"?

To make it clearer, we adapted the sentence as follows:

"To ensure nonbiased quantification of immunosignals we used FIJI to automatically select foci in leptotene and zygotene nuclei, see Materials and Methods for details."

Line 156 Why did you not consider the possibility that the non-random peak at 800 nm represents the equivalent of the paired co-foci we observed at around 400 nm in budding yeast?

At 800 nm distance, paired co-foci should have been visible by standard immunofluorescence microscopy. However, the foci appear organized as beads on a string of SC. However, we certainly agree that the suggested possibility should be discussed in the manuscript and have added the following sentence:

"This could reflect a form of interference between DSBs, since the foci appear organized as beads along a string of SC. However, we also cannot exclude that some of these distant foci actually represent a pair of single ends of a DSB at a preferred distance of around 800 nm to each other, similar to the co-foci observed in yeast, at shorter distance (13). The fact that the peaks around 800 nm are somewhat more pronounced when the mask is not used may at least in part be caused by background signal. In addition, some foci may localise just outside the mask, away from the axes, at around 800 nm distance from foci on the axes, and contribute to the 800nm peak frequency."

Line 251 Don't you need to specify "paired" co-foci? i.e. "confirmed the notion that **paired** RAD51-DMC1 co(nano)foci are rare"

We agree with the suggestion, and adapted the manuscript accordingly

Line 311-314. I think this passage is still unclear. I think you are trying to say something like "the greater distance between the two centers of mass of RAD51-RAD51 (or DMC1-DMC1) nano-focus pairs, as compared to the distance between the two centers of mass of adjacent Rad51-Dmc1 pairs, truly reflects greater spatial separation and cannot be accounted for by elongated focus shape alone."

We apologize that we still have not been able to phrase this section in a more comprehensive manner. The problem is that lines 311-314 refer to all DxRy foci with x or y >1. So these may have multiple different distances between centers of mass among RAD51 and DMC1 nanofoci, but also between RAD51 and DMC1 nanofoci. Therefore, we feel we should not rephrase this general section as suggested. To make it clearer, we now only state that these foci always have a larger distance, and leave the distinction between elongated foci and actual larger distance between nanofoci to the next paragraph as follows:

"Since only a single nanofocus is present for each of the individual recombinases in the D1R1 group, the distribution of the maximum distance was the same as for the minimum distance. Importantly, it completely overlapped with the first peak of the distribution of maximum distances of all configurations. This shows that all foci with more than one RAD51 and/or DMC1 nanofocus, always have at least one RAD51-RAD51, DMC1-DMC1, or RAD51-DMC1 nanofoci distance that is larger,

with an average distance of around 300 nm (wild type/*Sycp1*$^{-/-}$;287.4±2.7 SEM/308.6±2.8 SEM (Fig 5B).This observation of asymmetry allowed us to define close and far nanofoci in both the D2R1 and the D1R2 configurations. Interestingly, we observed a large close nanofocus and a small far nanofocus irrespective of whether two RAD51 or two DMC1 nanofoci were present (Fig5C-E). Thus, the measured larger distance between the two DMC1 or RAD51 nanofoci in the D2R1 and D1R2 configurations can be interpreted as more spatial separation, and is not caused by a second structure that is very spread out in its localisation pattern (since the far nanofocus is always relatively small)."

Line 463. See comment re Line 156. You do seem to entertain the possibility that the two ends could be farther apart than 300 nm, but why then is the 800 nm peak not a candidate for truly paired co-foci?

As indicated above, we agree that the 800nm peak could be viewed as a pair of cofoci in some cases, but we do not think this is a general feature, since this would have then been obvious from just viewing the immunofluorescent microscopic images. In addition, the foci are usually visible as a chain with odd or even numbers, not in pairs. However, we do agree that because of the fact that different foci may represent different stages of repair, and because there is of course variability in foci distances, clear pair formation may not be visible, but still present. To put more emphasis on this possibility we have rephrased this section as follows:

"We hardly observed configurations that could be considered to be paired D1R1 nanofoci, contrary to what might be expected based on observations in yeast (13), and from the symmetric loading of DMC1 observed in ChIP-seq data of mouse meiotic hotspots (32). Also, in the nematode *C. elegans*, where chromosome pairing precedes meiotic DSB formation, the majority of RAD51 foci could be resolved as paired structures using structured illumination microscopy (33). However, it might be suggested that if D1R1 configurations represent a single end of the DSB, the distance to a paired other DSB (D1R1) end would be on average 800nm (based on the nearest neighbour analysis), but still highly variable, precluding visible paired occurrences D1R1 foci. This could then be considered analogous to the paired co-foci observed in yeast and *C. elegans* (13, 33). Alternatively, the other DSB end could also be occupied by other ssDNA binding proteins, while a combination of these two configurations may also occur."

Line 535-539. We proposed that more than one focus could occupy a ssDNA tract on one end of a DSB given the small size of foci relative to the predicted length of ssDNA tracts. Are you excluding this possibility for some reason?

We do not exclude this possibility and agree it should be discussed in the manuscript. We did not consider this because the multiple nanofoci in a single ROI are spatially separated from each other, which does not immediately suggest presence of these foci on the same ssDNA. We have adapted this section as follows (including mentioning of the possibility of paired foci occurrence also in this concluding section):

"Our super-resolution dual colour dSTORM approach allowed direct comparison of the localization of RAD51 and DMC1 relative to each other. We provide the first evidence for the presence of a major structure consisting of a single relatively large nanofocus of both RAD51 and DMC1 in close proximity to each other in the majority of mouse meiotic DSB repair foci. Additional, smaller nanofoci of either recombinase are often present, and the fact that the total number of non-overlapping nanofoci exceeds two in ~20% of the foci indicates that either multiple non-overlapping nanofoci occupy different segments of ssDNA, as suggested by Bishop et al., (13), or some nanofoci represent binding to dsDNA, or chromatin, or background, since maximally two DSB ends are expected to be available for binding within a single ROI. We favour the hypothesis that the D1R1 configuration mostly represents formation of two adjacent filaments of RAD51 and DMC1 on the same molecule. This then suggests that one DSB end is often not bound by the recombinases, or epitopes are hidden due to differential conformations of the two ends, or the two ends are paired but far apart (average 800 nm), with a wide variety in distances, precluding visible formation of paired co-foci."

Fig 2F. I apologize that I did not examine the high-resolution version of this figure as carefully as I might have on the first submission. Now that I am looking carefully at the high res version of the figure, I see what you were talking about regarding “clusters.” For example, many D1R1s have multiple distinct maxima for at least one of the two immuno-stains. I appreciate that the approach of converting to binary images was needed to reduce the complexity of the images to a form that allowed you to place ROIs into categories, but clearly a lot of detail was lost as a result of the conversion. I think a few sentences should be added to the discussion to highlight this issue—i.e. that the method of analysis simplified the structural complexity of ROIs significantly, but the simplification was required to provide a means to detect differences in focus configurations between stages and genotypes.

We appreciate this comment., It should be noted that the representation of dSTORM data in Figure 2F was made by plotting Gaussian distributions for all localizations with a width corresponding to their precision, which is the most common method to visualize dSTORM data. This results in an image-like representation of the data where small dots represent better localized molecules and more blurred dots are less precisely localized molecules. This makes interpretation less straightforward compared to the regular “what you see is what you get”-like microscopy images. We agree that the clustered and binary images, indeed simplify the data but also represent an easier and more realistic interpretation of the data in that the obtained areas more closely correspond to the actual area where the molecules were present. To make this clearer, we adapted the discussion as follows:

“RAD51 and DMC1 filaments are expected to form elongated structures, based on super-resolution images of RAD51 in somatic cells (26, 27). The maximal length of the RAD51 and DMC1 nanofoci in all observed configurations reached an average of around 140 nm in pachytene, based on our simulations, but the maximal length of the most stretched RAD51 or DMC1 nanofoci was found to be around 250-300 nm. This is comparable to the previously observed elongated RAD51 structures in fixed somatic cells using dSTORM (26). Haas et al. (27) observed an average maximal length of RAD51 nanofoci of around 160 nm, similar to the average simulated length we observed. It should be noted that we performed our analysis on binary images, where clusters are generated from the kernel density plots of each ROI, with a threshold at a specific localization density. In doing so, we reduced complexity and inevitably lost some information. However, this approach allowed systematic comparison of the results between images in the same meiotic stage as well as in different stages. Although there might be some internal structure visible in the foci images shown in Fig. 2F, it should be noted that the precision of each localisation event varies, and affects the gaussian distribution-based representation of the dSTORM data. In addition, the resolution is limited by the sizes of the primary and secondary antibodies, which is expected to add around 20-40 nm in X and Y direction in our 2D images (28, 29). Moreover, variability in binding may influence the actual “shape” generated by the gaussian distribution generated from the localisation events coming from the fluorophores attached to the secondary antibodies. Therefore, we feel that the binary images provide an optimal representation of the actual areas covered by the recombinases and bound antibodies.”

Also, I think the way Fig 2F is designed is awkward. Rather than having a color-coded boundary on different parts of the figure, it would be simpler to separate the groups of examples with a white border and place a label above each group.

We thank the referee for this suggestion and adapted the figure in the revised version

What are the examples below the blue border that have no designation currently?

we apologize for this unclarity. These examples represent all other configurations, which has now been clearly indicated in the revised manuscript.

Same issues apply for Sup Figure 3 E.

This figure has now also been adapted as suggested

Fig 6. The use of standard error in the plot in C obscures the large range of size measurements represented in B. I wonder if a scatter plot or some other method of data presentation would be more informative regarding the obviously wide range of axis-to-focus-center measurements.

We thank the referee for this useful comment and have replaced the bar graphs with box plots that provide more detail and indeed more clearly show the large range of size measurements in these experiments. The legend and Materials and Methods have been adapted accordingly.

Sup Fig 2C. Eliminate the redundant presentation of the data resulting from the symmetry of the table format. Boxes on and above the diagonal should be eliminated as should the P column. Same change for Sup Figure 3C; there are only 3 comparisons in that table.

This has now been corrected as suggested.

Response to Reviewer 1 comments:

The new analysis measuring the distances of foci to the axis is a nice addition to the work-here it would be more informative to represent the range of distance measurements rather than a simple bar graph with SEM error bars – at a minimum, use SD for error bars, but best to use either box and whiskers or vertical scatter plot so the full distribution and range of distances can be visualized.

We thank the reviewer for the suggested improvement and have adapted the graphs as suggested.

The more complete description of the method for generating random distributions for Figs 1 and S1 is appreciated. I also like the solution the authors chose to address the reasons for and possible complications caused by using a mask, i.e., by presenting nearest-neighbor analyses conducted both with and without an Sycp3 mask. The only remaining comments I have are in regards to the presentation and outcomes of these analyses:

In the legend for Fig 1, please state that the distance distributions are for foci associated with Sycp3 masked regions.

This has now been included in the revised legend

In the legend for Fig S1, please state that the distance distributions are for all foci within the analyzed nuclei.

This has now been included in the revised legend

I am struck by the fact that the sharp peaks for D-D and R-R nearest neighbor distances at 700-800 nm not only persisted when all foci were analyzed, but actually increased (as a fraction of total distances). I appreciate that the authors are being judiciously circumspect about interpreting the possible basis for the 800 nm peaks, but this finding does warrant further discussion. The fact that this feature was not dampened, but instead emphasized in the "all foci" analysis suggests that use of the axis mask was partially obscuring the effect. This in turn raises the possibility that some axis associated foci might have a corresponding partner focus located out in a loop and/or extended along the axis, and/or reaching out to the homolog

– perhaps the other side of the DSB? The 700-800 nm distance is clearly longer than the typical distances between the paired foci observed in yeast or worms, but is it really ruled out that they could represent partner foci? At a minimum, this issue needs to be discussed, and may affect how some statements are made in the abstract.

This is a very interesting point, also raised by the other reviewer. Regarding the dampening of the 800 nm peak upon using the mask, this can in part be explained by loss of some background 800 nm pairs, but we agree that it is also very well possible that the use of the mask obscured some true 800 nm interactions because some foci may have been away from the axes.

We have adapted a section in the abstract as follows:
"D2R2 foci were rare (<10%) and nearest neighbour analyses also did not reveal co-foci formation between D1R1 foci. However, overall, foci localised non-randomly along the SC, and the frequency of the distance distributions peaked at 800nm, indicating interference and/or a preferred distance between two ends of a DSB."

Other sections that have been adapted with respect to the nearest neighbour analyses (also in response to the similar associate editors comment) are again pasted below:

Results:

"This could reflect a form of interference between DSBs, since the foci appear organized as beads along a string of SC. However, we also cannot exclude that some of these distant foci actually represent a pair of single ends of a DSB at a preferred distance of around 800 nm to each other, similar to the co-foci observed in yeast, at shorter distance (13). The fact that the peaks around 800 nm are somewhat more pronounced when the mask is not used may at least in part be caused by background signal. In addition, some foci may localise just outside the mask, away from the axes, at around 800 nm distance from foci on the axes, and contribute to the 800nm peak frequency."

Discussion:
"We hardly observed configurations that could be considered to be paired D1R1 nanofoci, contrary to what might be expected based on observations in yeast (13), and from the symmetric loading of DMC1 observed in ChIP-seq data of mouse meiotic hotspots (32). Also, in the nematode *C. elegans*, where chromosome pairing precedes meiotic DSB formation, the majority of RAD51 foci could be resolved as paired structures using structured illumination microscopy (33). However, it might be suggested that if D1R1 configurations represent a single end of the DSB, the distance to a paired other DSB (D1R1) end would be on average 800nm (based on the nearest neighbour analysis), but still highly variable, precluding visible paired occurrences D1R1 foci. This could then be considered analogous to the paired co-foci observed in yeast and *C. elegans* (13, 33). Alternatively, the other DSB end could also be occupied by other ssDNA binding proteins, while a combination of these two configurations may also occur."
